# Improving adoptive T cell therapy with adenoviruse armed with TNF-α and IL-2

**DOI:** 10.1186/2051-1426-3-S2-P41

**Published:** 2015-11-04

**Authors:** Riikka Havunen, Mikko Siurala, Suvi Parviainen, Simona Bramante, Dipongor Saha, Siri Tähtinen, Markus Vähä-Koskela, Michael Behr, Dirk Nettelbeck, Anja Ehrhardt, Akseli Hemminki

**Affiliations:** 1 Cancer Gene Therapy Group, Medicum, Haartman Institute, University of Helsinki, Helsinki, Finland, Helsinki, Finland; 2 TILT Biotherapeutics Ltd, Helsinki, Finland, Helsinki, Finland; 3 German Cancer Research Center (DKFZ), Heidelberg, Germany, Heidelberg, Germany; 4 Witten/Herdecke University, Witten, Germany, Witten, Germany

## Background

The field of T cell therapy of cancer holds great promise, as demonstrated by results obtained in Chimeric Antigen Receptor (CAR) modified T cell therapy of CD19+ leukemia. However, these anti-tumor effects are often limited by T cell tolerance and the immunosuppressive tumor microenvironment. Oncolytic viruses armed with immunostimulatory molecules constitute a potent form of immunotherapy. In essence, the danger signal caused by virus replication, coupled with the actions of the transgene, and effective presentation of tumor epitopes by lysis of the cells, results in a personalized cancer vaccine, manufactured for each patient in their tumor.

Previously, we have identified interleukin 2 (IL-2) and Tumor Necrosis Factor alpha (TNF-α) as the most promising factors to stimulate the graft used in adoptive T cell therapy. Both cytokines are important activators of immune cells and also known for their direct anti-tumor properties. Therefore, we aimed to enhance the efficacy of ACT by coupling it with adenoviruses coding for immunostimulatory cytokines. Notably, these cytokines can cause severe side effects when administered systemically. Previously, we and others have achieved long lasting, high level cytokine expression locally but low level systemically when using armed oncolytic viruses *in vivo*.

## Methods

Therefore, we developed oncolytic adenoviruses expressing one or both above mentioned human cytokines (Ad5/3-E2F-D24-hTNF-α, Ad5/3-E2F-D24-hIL-2 and Ad5/3-E2F-D24-hTNF-α-IRES-hIL-2). For more detailed immunological studies in immune competent mouse models, we constructed non-replicative adenoviruses with murine cytokines (Ad5-CMV-mIL-2 and Ad5-CMV-mTNF-α). These viruses were studied in combination with adoptive transfer of OT-1 TCR transgenic T cells to treat C57BL/6 mice bearing B16-OVA melanoma tumors. The animals were administered intraperitoneally with CD8+-enriched OT-1 cells with or without intratumoral injections of cytokine-coding viruses.

## Results

Combination treatment with Ad5-CMV-mIL-2 and OT-1 resulted in statistically significant anti-tumor efficacy when compared with either monotherapy or untreated control. In further experiments a triple combination of Ad5-CMV-mIL-2 + Ad5-CMV-mTNF-α and OT-1 T cells improved anti-tumor efficacy was observed over dual agent therapies. Furthermore, in mice bearing two tumors we saw abscopal effect when only one of the tumors was injected with cytokine encoding viruses but reduction in tumor size was detected also in the non-injected tumor.

## Conclusions

In summary, these results support the further development of cytokine-armed adenoviruses as facilitators of adoptive T cell therapy.

